# Sleep and Cytokines: A Bidirectional Dialogue Involving Rest and Immunity

**DOI:** 10.3390/children13040535

**Published:** 2026-04-12

**Authors:** Ignazio Cammisa, Margherita Zona, Giorgia Petracca, Eleonora Rulli, Chiara Veredice, Clelia Cipolla, Donato Rigante

**Affiliations:** 1 Department of Pediatrics, San Giovanni Evangelista Hospital, 00019 Tivoli, Italy; 2 Department of Life Sciences and Public Health, Fondazione Policlinico Universitario A. Gemelli IRCCS, 00168 Rome, Italy; margherita.zona01@icatt.it (M.Z.); eleonora.rulli01@icatt.it (E.R.); clelia.cipolla@policlinicogemelli.it (C.C.); donato.rigante@unicatt.it (D.R.); 3 Pediatric Neurologic Unit, Department of Woman and Child Health and Public Health, Fondazione Policlinico Universitario A. Gemelli IRCCS, 00168 Rome, Italy; giorgia.petracca01@icatt.it (G.P.); chiara.veredice@policlinicogemelli.it (C.V.); 4 Periodic Fevers Research Center, Università Cattolica Sacro Cuore, 00168 Rome, Italy

**Keywords:** sleep regulation, cytokines, inflammation, immunity, personalized medicine, innovative biotechnologies

## Abstract

**Highlights:**

**What are the main findings?**

- Sleep and the immune system are tightly interconnected through cytokine-mediated signaling, with interleukin-1β, tumor necrosis factor-α, and interleukin-6 acting as seminal regulators of sleep architecture and buildout of inflammatory responses.
- Sleep deprivation and sleep disorders may alter cytokine production and circadian rhythmicity, promoting systemic inflammation and contributing to immune, metabolic, and neuroendocrine dysregulation.

**What are the implications of the main findings?**

- Dysregulated cytokine signaling represents a mechanistic link between sleep disturbances and increased susceptibility to inflammatory, metabolic, and neuropsychiatric conditions.
- Targeting sleep quality and inflammatory pathways, including cytokine modulation, might offer therapeutic opportunities to improve both sleep regulation and immune homeostasis.

**Abstract:**

Sleep is a cardinal biological process that backstops central nervous system function, which also plays a crucial role in regulating systemic homeostasis, including immune activities. Cytokines, particularly interleukin-1β and tumor necrosis factor-α, act as mediators bridging sleep and inflammation, also influencing both sleep architecture and sleep–wake cycle. Sleep deprivation and sleep disorders such as insomnia, narcolepsy, hypersomnia, or obstructive sleep apnoea may disrupt cytokine production, alter their circadian rhythm of release, and shift secretion peaks from night to day. These changes contribute to daytime fatigue, impaired cognitive and physical performance, increased susceptibility to infections and/or systemic inflammation. Molecular studies indicate that insufficient sleep primes immune cells to enhance pro-inflammatory responses, creating a feedback loop with neuroendocrine pathways that further exacerbates sleep patterns and inflammatory dysregulation. Understanding the bidirectional relationship between sleep and cytokines may highlight the role of sleep as an active component of immunity regulation and underscore the potential usefulness of multilevel interventions that include complementary and integrative health approaches restoring sleep, normalizing cytokine rhythms and mitigating inflammation.

## 1. Introduction

**Figure 1:**
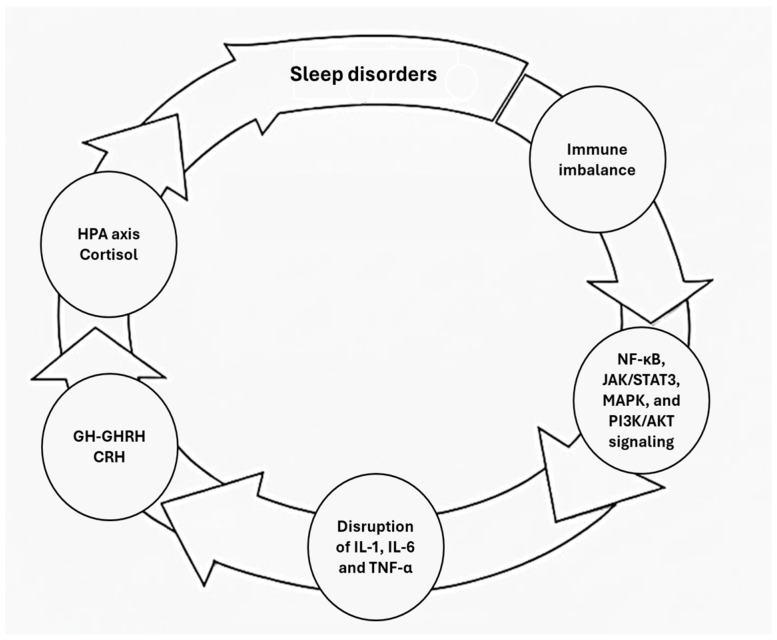
Bidirectional sleep–immune–neuroendocrine interactions in sleep disorders. Corticotropin-releasing hormone (CRH), Growth hormone (GH), Growth hormone-releasing hormone (GHRH), Hypothalamic–pituitary–adrenal axis (HPA), Interleukin-1 (IL-1), Interleukin-6 (IL-6), Janus kinase/Signal transducer and activator of transcription 3 (JAK/STAT3), Mitogen-activated protein kinase (MAPK), Nuclear factor kappa-light-chain-enhancer of activated B cells (NF-κB), Phosphoinositide 3-kinase/Protein kinase B (PI3K/AKT), Tumor necrosis factor (TNF).

Sleep occupies approximately one-third of the human lifespan and represents a fundamental biological process essential for physiological recovery, adaptation, and survival. Historically, sleep was primarily regarded as a state supporting central nervous system (CNS) functions, including memory consolidation, emotional enrichment, and cognitive performance [1,2]. Over the past decades, however, experimental studies have redefined sleep as a pivotal regulator of systemic homeostasis, with profound effects on metabolic balance, cardiovascular function, endocrine regulation, and immune competence [3,4,5,6]. Large-scale epidemiological and longitudinal studies consistently show that insufficient sleep duration, sleep fragmentation, or poor sleep quality is associated with increased morbidity and mortality. Chronic sleep disturbances have been linked to heightened susceptibility to infectious diseases, impaired vaccine responses, exacerbation of inflammatory and autoimmune conditions, and an increased risk of immune-mediated disorders [7,8,9,10]. These findings highlight a tightly integrated reciprocal relationship between sleep and the immune system, mediated by a complex network of cytokines and inflammatory signaling molecules. The regulation of sleep involves neuronal circuits, glial cells, immune mediators, and cytokine-driven signaling pathways. Conversely, alterations in sleep duration or quality can profoundly affect immune function, particularly innate responses, by modulating cytokine release and inflammatory tone [11,12,13]. Among these mediators, pro-inflammatory cytokines such as interleukin-1β (IL-1β) and tumor necrosis factor-α (TNF-α) play an active role in linking immune activation with sleep regulation, crucially contributing to the initiation, maintenance, and intensity of non-rapid eye movement (NREM) sleep. In contrast, interleukin-6 (IL-6) is primarily involved in the regulation of rapid eye movement (REM) sleep [11]. Collectively, sleep patterns influence immune function by regulating leukocyte trafficking and orchestrating effective innate and adaptive responses [10,11,12,13]. This evidence supports the notion that sleep is an integral component of immunity regulation, critically mediated by cytokine signaling. This review aims to synthesize the current evidence on the bidirectional relationship between sleep and cytokine production, as illustrated in Figure 1.

## 2. Cytokines in Immune and Inflammatory Signaling

**Figure 2:**
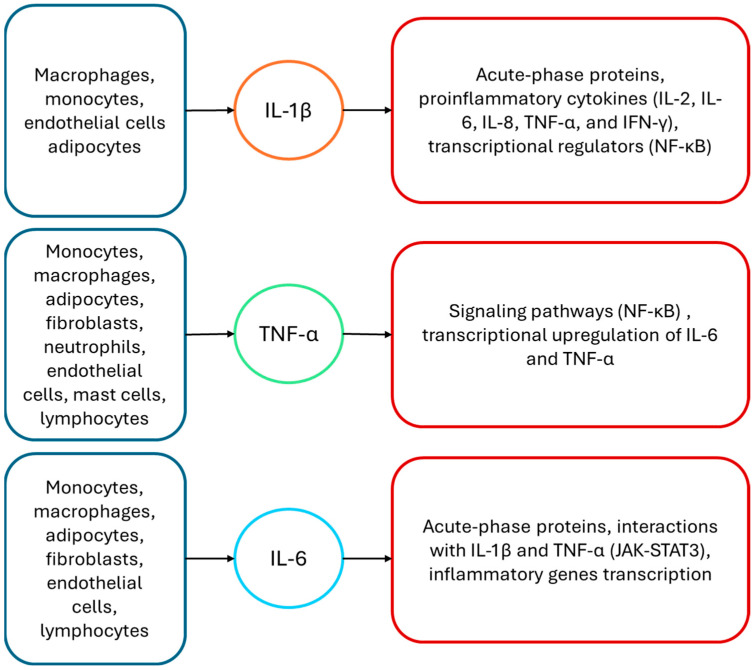
Main features of the pro-inflammatory cytokines: interleukin-1β, tumor necrosis factor-α and interleukin-6. Interferon-gamma (IFN-γ), Interleukin-1β (IL-1β), Interleukin-2 (IL-2), Interleukin-8 (IL-8), Interleukin-6 (IL-6), Tumor necrosis factor (TNF), Janus kinase/Signal transducer and activator of transcription 3 (JAK/STAT3), Nuclear factor kappa-light-chain-enhancer of activated B cells (NF-κB).

Cytokines comprise a heterogeneous group of low molecular weight signaling molecules, including ILs, colony-stimulating factors (CSFs), interferons (IFNs), and TNF: they are synthesized and released by a broad spectrum of immune and non-immune cell types such as leukocytes, endothelial cells, adipocytes, and glial cells [14]. Acting via autocrine, paracrine, and endocrine mechanisms, these cytokines orchestrate immune communication and play a relevant role in the regulation of systemic homeostasis [15,16,17]. According to their dominant biological activities, cytokines are commonly categorized as either pro-inflammatory or anti-inflammatory mediators. The pro-inflammatory cytokines, including IL-1β, TNF-α, and IL-6, are essential components of host defense, as they facilitate leukocyte recruitment, trigger febrile responses, and activate immune effector mechanisms during infections and different tissue damage. Conversely, anti-inflammatory cytokines, such as IL-10 and transforming growth factor-β, suppress excessive immune activation, prevent collateral tissue injury, and promote the resolution of inflammation [14,17,18]. The interplay between pro-inflammatory cytokines and their counter-regulatory ones constitutes a highly coordinated network that ensures a dynamic balance between effective immune responses and immune homeostasis [14]. Among pro-inflammatory mediators, IL-1β, TNF-α, and IL-6 have been extensively investigated and are recognized as regulators of both innate and adaptive immune responses, as reported in Figure 2.

IL-1β is generated primarily by activated macrophages, monocytes, endothelial cells, and adipocytes in response to danger-associated molecular patterns: its biological activity is mediated through binding to the type I IL-1 receptor (IL-1R1), which is expressed on a variety of cell types, including T lymphocytes, endothelial cells, and fibroblasts [14,19]. IL-1β stimulates hepatocytes to produce acute-phase proteins and induces local cells to secrete additional pro-inflammatory mediators such as IL-2, IL-6, IL-8, TNF-α, and IFN-γ. This feed-forward amplification mechanism substantially enhances inflammatory signaling. Furthermore, IL-1β activates nuclear factor kappa-light-chain-enhancer of activated B cells (NF-κB) and other transcriptional regulators, thereby reinforcing downstream chemokine expression within the inflammatory cascade [14,20]. TNF-α is a multifunctional glycoprotein predominantly produced by activated monocytes and macrophages, with additional contributions from adipocytes, keratinocytes, fibroblasts, neutrophils, endothelial cells, mast cells, and selected lymphocyte populations [19]: it mediates its biological effects through selective interaction with two cell-surface receptors, namely TNF-R1 and TNF-R2, which are expressed by nearly all nucleated cells. The activation of these receptors initiates intracellular signaling pathways, most prominently involving the NF-κB, resulting in the transcriptional upregulation of multiple pro-inflammatory genes, including IL-6 and TNF-α itself [14]. Furthermore, TNF-α is a critical determinant of host resistance to viral, bacterial, fungal, or parasitic infections and is particularly important in the immune response against intracellular pathogens such as *Mycobacterium tuberculosis* and *Listeria monocytogenes*. However, TNF-α dysregulated overproduction contributes to disease severity and leads to dangerous pathological conditions, including septic shock [17]. IL-6 is mainly produced by monocytes and macrophages, but also by T and B lymphocytes, fibroblasts, endothelial cells, and adipocytes: it transduces its signals via the IL-6 receptor (IL-6R), which activates the signal-transducing subunit gp130 that displays an intrinsic tyrosine kinase activity [14,18]. IL-6 also plays a central role in immune modulation by inducing acute-phase protein synthesis by the liver and promoting the differentiation of B cells into antibody-secreting plasma cells. In addition, IL-6 contributes to the initiation and persistence of inflammatory responses through positive feedback interactions with IL-1β and TNF-α. These effects are mediated in part by activation of the JAK/STAT3 signaling pathway, which cooperates with NF-κB to regulate inflammatory gene transcription [21]. Overall, these cytokines constitute a tightly interconnected signaling network that not only orchestrates innate and adaptive immune responses but also serves as a critical interface between inflammation and other physiological systems, thereby providing a mechanistic framework for understanding how immune activation can influence complex biological processes, such as sleep regulation.

## 3. Cytokines as Sleep-Regulatory Mediators

### 3.1. Circadian Variability of Cytokine Secretion

**Figure 3:**
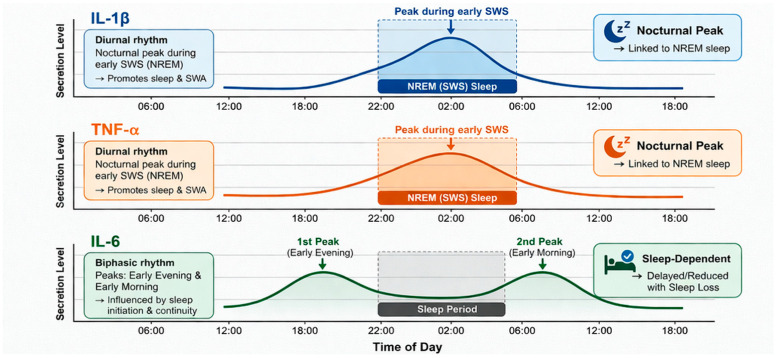
Circadian secretion profiles of pro-inflammatory cytokines during sleep. Interleukin-1β (IL-1β), interleukin-6 (IL-6), non-rapid eye movement (NREM), slow-wave sleep (SWS), tumor necrosis factor-α (TNF-α).

Pro-inflammatory cytokines exhibit characteristic circadian and sleep-dependent secretion profiles that are tightly coupled to sleep regulation, as illustrated in Figure 3 [1]. In particular, IL-1β and TNF-α display diurnal rhythms with nocturnal peaks that occur predominantly during the early slow-wave sleep (SWS)-dominated portion of the night and are closely linked to NREM sleep. Through their somnogenic properties, these cytokines promote sleep depth and slow-wave activity [22,23,24,25].

In contrast, IL-6 follows a biphasic rhythm with peaks in early evening and in early morning, and its nocturnal increase is strongly influenced by sleep initiation and continuity, being delayed or attenuated under conditions of sleep loss. Consequently, disturbances in sleep or circadian organization disrupt the normal temporal pattern of cytokine release, resulting in elevated daytime concentrations and blunted nocturnal peaks, underscoring the reciprocal relationship between sleep architecture, circadian regulation, and inflammatory signaling [1].

### 3.2. Direct Effects of Cytokines on Brain Networks

The mechanisms underlying the nocturnal elevation of cytokines remain incompletely understood. One hypothesis proposes that wakefulness is associated with the accumulation of danger-associated molecular signals, including reactive oxygen species, extracellular nucleotides (e.g., ATP), and heat-shock proteins (HSPs), which subsequently activate immune signaling pathways and stimulate cytokine production. Alternatively, cytokine levels may peak during the early SWS-dominated portion of sleep, coinciding with the release of growth hormone (GH) and prolactin, neuroendocrine factors that promote T-cell proliferation or differentiation, and type 1 cytokine activity [2,26]. The precise neural substrates and molecular mechanisms involved are not yet fully elucidated [24]. Experimental studies indicate that IL-1β and TNF-α directly modulate sleep–wake circuitry by acting within key NREM-regulatory regions, where they shift a balance between wake-promoting and sleep-active neuronal populations [27,28]. IL-1β acts on the dorsal raphe nucleus to inhibit serotonergic neuronal activity, while TNF-α exerts its effects in the preoptic area; both actions result in increased NREM sleep [27,28,29,30].

### 3.3. Neuroendocrine Modulation of Cytokine Effects

Cytokine effects on sleep are further mediated by neuroendocrine pathways, particularly growth hormone-releasing hormone (GHRH) and corticotropin-releasing hormone (CRH) signaling [24]. GHRH facilitates NREM sleep, whereas CRH promotes arousal; a shift toward CRH dominance is associated with fragmented sleep, elevated cortisol levels, and suppressed GH secretion. IL-1β influences sleep by stimulating both pathways, with its NREM-promoting effects mediated primarily through GHRH.

**Figure 4:**
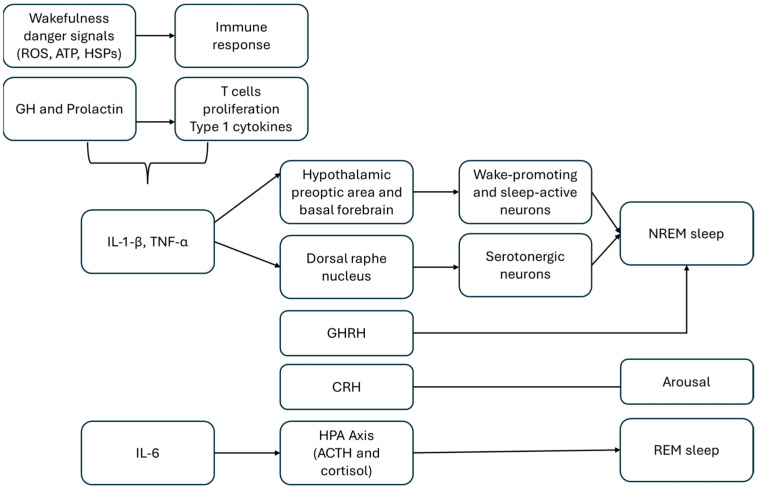
Mechanisms of cytokine-mediated regulation of sleep. Adrenocorticotropic hormone (ACTH), Adenosine triphosphate (ATP), Corticotropin-releasing hormone (CRH), Growth hormone (GH), Growth hormone-releasing hormone (GHRH), Interleukin-1 (IL-1), Interleukin-6 (IL-6), Heat shock proteins (HSPs), Hypothalamic–pituitary–adrenal (HPA) axis, Non-rapid eye movement (NREM) sleep, Rapid eye movement sleep (REM), Reactive oxygen species (ROS), Tumor necrosis factor (TNF).

The hypothalamic–pituitary–adrenal (HPA) axis provides feedback regulation. Specifically, HPA activity favors increased IL-1β expression and enhanced NREM sleep [28,29,30,31]. IL-6 interacts bidirectionally with the HPA axis, integrating immune and neuroendocrine responses to stress and inflammation [1,32]. Experimental and clinical evidence demonstrates that exogenous IL-6 administration stimulates HPA axis activity in humans, resulting in increased secretion of adrenocorticotropic hormone (ACTH) and cortisol. These findings suggest a direct activating influence of IL-6 on HPA axis dynamics, that is at least partially independent of classical hypothalamic CRH signaling [33]. Conversely, glucocorticoids exert negative feedback on IL-6 synthesis, thereby constraining inflammatory signaling under physiological conditions [34]. IL-6 also influences sleep architecture in a time- and context-dependent manner. Experimental studies, such as those by Redwine et al., show that IL-6 administration alters sleep structure, including a reduction in SWS and changes in REM sleep parameters, without consistently affecting total NREM sleep. Elevated IL-6 levels during the evening and early night are associated with HPA axis activation and increased cortisol secretion, thereby promoting arousal and impairing sleep initiation and continuity [11,32]. In humans, sleep loss is linked to excessive daytime IL-6 secretion, which inversely correlates with SWS and negatively affects sleep quality and daytime well-being [11,32]. Conversely, SWS is characterized by reduced HPA axis activity and lower cortisol levels, creating a physiological ‘milieu’ that limits IL-6 production and supports immune–neuroendocrine homeostasis. Disruption of this balance through sleep deprivation or fragmentation leads to sustained IL-6 elevation and persistent HPA axis activation, reinforcing a maladaptive feedback loop between inflammation, stress, and impaired sleep regulation [1,11,32]. The main mechanisms discussed herein are summarized in Figure 4.

## 4. Sleep Disorders and Cytokine Dysregulation

### 4.1. Evidence on Sleep and Cytokine Dysregulation from Studies in Adults

Sleep disorders are increasingly recognized as conditions characterized by profound immune and inflammatory dysregulation, with multiple cytokines involved in their pathophysiology and systemic effects. Experimental studies manipulating sleep duration—through total or partial sleep deprivation and chronic sleep restriction—have consistently demonstrated these effects [1]. Prolonged wakefulness increases IL-1β–like activity, while TNF-α levels rise rapidly after 1–2 nights of total or partial sleep deprivation. Chronic sleep restriction likewise elevates TNF-α and IL-1β transcript levels, with effects that may persist even after recovery sleep. Notably, individuals with pre-existing sleep disturbances exhibit heightened TNF-α responses even after a single night of sleep loss, reflecting increased immune vulnerability under conditions of chronic sleep impairment [1,35,36,37,38,39]. Sleep loss also alters the diurnal pattern of inflammatory mediators. IL-6 levels increase during the daytime and decrease at night, especially in association with changes in sleep stages 1, 2, and REM relative to slow-wave sleep (SWS) [1,39]. However, cumulative inflammatory responses—characterized by sustained increases in IL-6 and C-reactive protein—require longer periods of sleep restriction to become evident [36,37,38].

Chronic insomnia is one of the most prevalent conditions and is associated not only with elevated daytime levels of TNF-α and IL-6, but also with increased cortisol and CRH levels, contributing to fatigue, sleepiness, and poor sleep quality [40,41,42,43]. In individuals with insomnia, the circadian pattern of IL-6 is altered, with its primary peak shifting from early morning to the evening, while TNF-α rhythmicity is blunted and replaced by shorter (~4-h) oscillations of significant amplitude. These alterations may contribute to impaired sleep–wake regulation and increased daytime somnolence. Similarly, patients with narcolepsy or hypersomnia exhibit higher plasma levels of TNF-α and IL-6 compared with matched controls [41]. Table 1 summarizes the main sleep disorders associated with alterations in cytokine profiles.

### 4.2. Cytokine Imbalance in Childhood Sleep Disorders

Emerging evidence suggests that sleep disorders in children are associated with alterations in inflammatory signaling, reflecting a dysregulation of cytokine activity. In a longitudinal analysis from the EDEN French birth cohort of 687 children, shorter and more variable sleep duration trajectories between ages 2 and 5 years were associated with higher circulating pro-inflammatory cytokines. Specifically, shorter sleep was linked to elevated IL-6 levels, while irregular sleep patterns were associated with increased TNF-α, independent of maternal and child-related confounding factors [35]. Similarly, the HELENA cross-sectional study in adolescents reported that shorter sleep duration was associated with higher cortisol levels and increased counts of white blood cells, neutrophils, monocytes, and CD4+ cells. In girls, shorter sleep was also linked to higher IL-5 and IL-6 levels. The study concluded that adolescents sleeping 8–8.9 h per night exhibited a more favorable immune profile, characterized by higher IL-4 and lower pro-inflammatory cytokine levels [42]. According to these findings, narcoleptic children, compared to healthy controls, exhibited significantly elevated levels of IL-6, IL-2, IL-4, TNF-α, IFN-γ, and IL-17 [43]. Other studies, although conducted in children with autism spectrum disorders, have shown that salivary IL-1β concentrations are inversely associated with sleep quality [44].

The majority of studies examining sleep disorders and cytokine profiles in pediatric populations have focused on obstructive sleep apnoea (OSA), in which both intermittent hypoxia and hypercapnia induce systemic inflammation via hypoxia-inducible factor-1 (HIF-1) and NF-κB pathways [43,44,45]. Children with OSA frequently have higher levels of IL-6, IL-8 and TNF-α, which improve after adenotonsillectomy [46,47,48,49]. TNF-α circadian rhythm is disrupted, with afternoon peaks replacing the normal nocturnal peak; IL-1β is markedly elevated, while IL-6 peaks in the evening rather than in early morning, indicating altered timing and magnitude of this pro-inflammatory cytokine secretion [50]. A meta-analysis of 63 studies (including six in children) showed that individuals with OSA have significantly higher circulating IL-6 levels compared to healthy controls. While this association was robust in adults (both serum and plasma IL-6), in children a significant increase was observed only for plasma IL-6 [47]. Few studies have found an association between cytokine levels and OSA severity. For example, Alexopoulos et al. reported TNF-α levels of 0.63 ± 0.2, 0.65 ± 0.18, and 0.63 ± 0.17 pg/mL in children with mild, moderate, and severe OSA, respectively, indicating that higher TNF-α levels cannot be used to predict OSA severity, while Nosetti et al. reported no differences in IL-1β production [49,51]. On the other hand, Gozal et al. found that morning TNF-α levels were globally increased in the presence of OSA, particularly in the most severe cases, and correlated with obstructive apnoea-hypopnea index and sleep pressure score, a measure of respiratory-induced sleep fragmentation. Additionally, morning TNF-α levels decreased after adenotonsillectomy in 22 children [52]. Although adenotonsillectomy significantly reduces the severity of pediatric OSA and attenuates systemic inflammation, the extent to which anatomical factors, such as tonsillar hypertrophy and adenoidal–nasopharyngeal ratio, modulate the inflammatory response to surgery remains unclear [47,49,51,52,53,54]. Likewise, it is not yet fully established whether baseline systemic inflammatory status influences post-operative OSA outcomes; probably tonsil monocytes, macrophages, and dendritic cells may have a role [48]. Molecular studies further support these findings: DNA microarray analyses in individuals experiencing three consecutive nights of insufficient sleep revealed upregulation of monocyte pro-inflammatory cytokines, with IL-6 mRNA increasing three-fold and TNF-α mRNA doubling [55].

## 5. Therapeutic Implications

Cytokine-targeted therapies have transformed the management of chronic inflammatory diseases through the selective inhibition of key pro-inflammatory mediators: these agents are widely employed in conditions such as rheumatoid arthritis, spondyloarthritis, and autoinflammatory syndromes, where they significantly improve clinical outcomes and patients’ overall quality of life [56,57,58,59]. Beyond their established anti-inflammatory effects, cytokine-targeted therapies have increasingly attracted interest for their potential impact on neuroimmune interactions, including sleep regulation [60,61,62]. The effects of anti-TNF-α therapy on sleep have been most extensively studied in patients with rheumatoid arthritis. In a prospective observational study, Karatas et al. showed that TNF-α inhibition improved subjective sleep quality and reduced fatigue, likely due to decreased systemic inflammation and pain [63]. Similarly, Taylor-Gjevre et al. reported that, after initiating anti-TNF-α therapy, polysomnography revealed significant improvements in objective sleep parameters, with sleep efficiency rising from 73.9% to 85.4% and wake time after sleep onset decreasing from 84.1 to 50.7 min [64]. In ankylosing spondylitis, anti-TNF-α therapy improved subjective sleep quality after three months, although no significant changes were seen in polysomnographic measures [65]. The potential effects of anti-IL-6 and anti-IL-1 therapies on sleep have been less extensively studied. Fragiadaki et al. found that the IL-6 receptor antagonist tocilizumab improved sleep quality and reduced daytime sleepiness in rheumatoid arthritis patients as early as one month after treatment, with further benefits over six months, alongside reductions in disease activity, fatigue, and improved functional status [66]. To date, no clinical studies have specifically assessed the effects of anti–IL-1 therapies, such as anakinra or canakinumab, on sleep as a predefined primary endpoint. Experimental research in healthy subjects, however, has shown that IL-1 receptor antagonism with anakinra can modify EEG parameters, enhancing slow-wave and NREM sleep [67]. Overall, while cytokine-targeted therapies show promise for improving sleep in adults with inflammatory diseases, no studies have evaluated the mechanisms of their effects on sleep in children, highlighting an important gap for future research.

## 6. Limitations

Despite substantial advances in understanding the bidirectional relationship between sleep and cytokine regulation, several limitations must be acknowledged. First, much of the evidence is extrapolated from adult studies, due to the relative scarcity of large-scale longitudinal pediatric research. Second, variability in study design and cytokine sampling times limits the direct comparability of findings across studies. Third, most investigations are observational, which precludes definitive conclusions about causality between sleep disturbances and cytokine dysregulation. Finally, the effects of interventions targeting cytokine pathways on sleep in children remain largely unexplored, restricting our ability to translate experimental adult findings into pediatric clinical practice. Future studies should prioritize longitudinal designs, standardized biomarker assessments, and interventional trials to clarify mechanisms, causality, and therapeutic potential, especially in the pediatric population.

## 7. Conclusive Remarks

Emerging evidence underscores a bidirectional relationship between sleep and cytokines. Sleep disturbances are associated with elevated pro-inflammatory cytokines such as IL-1β, TNF-α, and IL-6, often accompanied by alterations in their circadian secretion patterns. These changes can contribute to systemic inflammation or immune dysregulation and potentially have long-term health consequences. However, the precise mechanisms linking sleep, inflammation, and neuroendocrine signaling remain incompletely understood. Importantly, most evidence is extrapolated from adult studies, and interventional trials targeting cytokine pathways in pediatric populations are lacking. Overall, the findings discussed in this review highlight the critical need for well-designed longitudinal and interventional studies in children to clarify causal pathways, optimize therapeutic strategies, and understand the developmental implications of sleep–immune interactions. Recognizing and addressing cytokine dysregulation in pediatric sleep disorders may provide a pivotal opportunity to improve both short- and long-term health outcomes.

## Figures and Tables

**Table 1:** Association between altered cytokine profile and major sleep disorders.

Sleep Disorder	Alterations in Cytokine/Hormone Secretion
Chronic insomnia	↑ TNF-α and ↑ IL-6 during daytime; ↑ CRH and cortisol across 24 h
Narcolepsy	↑ TNF-α and ↑ IL-6; ↑ GH
Primary hypersomnia	↑ TNF-α and ↑ IL-6
Obstructive sleep apnoea	↑ TNF-α (afternoon peak), ↑ IL-1β, ↑ IL-6 (evening peak)

Tumor necrosis factor (TNF), Interleukin-6 (IL-6); Corticotropin-releasing hormone (CRH), Growth hormone (GH), Interleukin-1 (IL-1), increased level of (↑).

## Data Availability

No new data were created or analyzed in this study.
